# Usefulness of Ultrasound Examination in the Assessment of the Nail Apparatus in Psoriasis

**DOI:** 10.3390/ijerph19095611

**Published:** 2022-05-05

**Authors:** Magdalena Krajewska-Włodarczyk, Agnieszka Owczarczyk-Saczonek

**Affiliations:** 1 Department of Rheumatology, School of Medicine, Collegium Medicum, University of Warmia and Mazury, 10-900 Olsztyn, Poland; 2 Department of Dermatology, Sexually Transmitted Diseases and Clinical Immunology, School of Medicine, Collegium Medicum, University of Warmia and Mazury, 10-900 Olsztyn, Poland; aganek@wp.pl

**Keywords:** nail psoriasis, ultrasonography, psoriasis assessment

## Abstract

The assessment of psoriatic nail changes in everyday practice is based exclusively on clinical symptoms that do not reflect the entire disease process in the nail apparatus. The use of imaging methods, especially widely available and inexpensive ultrasonography, creates the possibility of additional revealing and assessing grayscale of morphological changes of the ventral nail plate, nail bed, and matrix, as well as the attachment of the finger extensor tendon to the distal phalanx. What is more, it enables the assessment of inflammation severity in the power Doppler technique. A qualitative classification of nail plate morphological changes corresponding to the severity of psoriatic nail changes has been developed so far and attempts are being made to develop a quantitative method to assess not only the presence of changes but also the severity of inflammation. Nail ultrasonography is not commonly performed, although published studies indicate the possible use of this technique in the assessment of psoriatic changes in nail structures. It can be particularly useful in subclinical changes imaging, preceding clinical manifestation of psoriatic nail changes, enthesopathy: subclinical and in the course of psoriatic arthritis, as well as in the assessment of treatment efficacy. This review article aims to summaries the research on ultrasonography of the nail apparatus which has been carried out so far, taking into account its applicability in clinical practice.

## 1. Introduction

Nowadays, healthy nails not only have a functional purpose of the hand, but they also have aesthetic value. Nail changes, similarly to skin lesions, are associated with a reduced quality of life. One of the clinical manifestations of psoriasis (Ps) is nail psoriasis, which, apart from the typical disfiguring changes, is associated with pain and significant functional disturbances. In addition, nail involvement is one of the risk factors for the development of psoriatic arthritis (PsA) [1], particularly involving the distal interphalangeal joints [2].

Psoriatic nail changes are present in approximately 10–55% of patients with psoriasis, but the risk of the occurrence of such changes during a patient’s lifetime may be as high as 80–90% [3]. Anatomical conditions may explain the extension of inflammation associated with nail psoriasis to adjacent structures, including the distal interphalangeal joints and finger extensor tendons [4]. It is also known that enthesopathies are one of the characteristic features of psoriatic arthritis [5,6]. The assessment of psoriatic nail changes in practice is mainly based on clinical assessment indices, such as the nail psoriasis severity index (NAPSI,) and the modified NAPSI index (mNAPSI) [7,8]. Ultrasonography (US) [9] and magnetic resonance imaging (MRI), mainly used for imaging of articular lesions [10], are available but rarely performed imaging methods in the assessment of the nail apparatus. MRI, due to its limited availability and high cost, is certainly not the examination of the first choice in nail imaging, whereas ultrasonography, as a widely available, non-invasive, and relatively inexpensive method, seems promising in assessing the progression of psoriatic nail changes, inflammatory changes of surrounding structures and treatment effects. High-resolution ultrasonography can be used as a measure for the morphological assessment of nail apparatus components and adjacent structures—on the scale of grayness and inflammatory activity—by assessing the severity of vascularization using the power Doppler (PD) technique [11,12,13].

## 2. Methods

A review of the literature was done to review the technical and clinical aspects of nail unit ultrasound. Using the PubMed database, we reviewed the literature on the use of ultrasound in the diagnosis and assessment of nail psoriasis. We used a combination of the keywords “nail psoriasis” together with “ultrasound”. Our search covered the period from 2000 up to April 2022. No formal inclusion and exclusion criteria were applied—all types of articles were included if deemed appropriate. Articles that described qualitatively and/or quantitatively the ultrasound examination of the anatomical structures of the nail apparatus were considered appropriate.

## 3. The Structure of the Nail Apparatus

**Figure 1:**
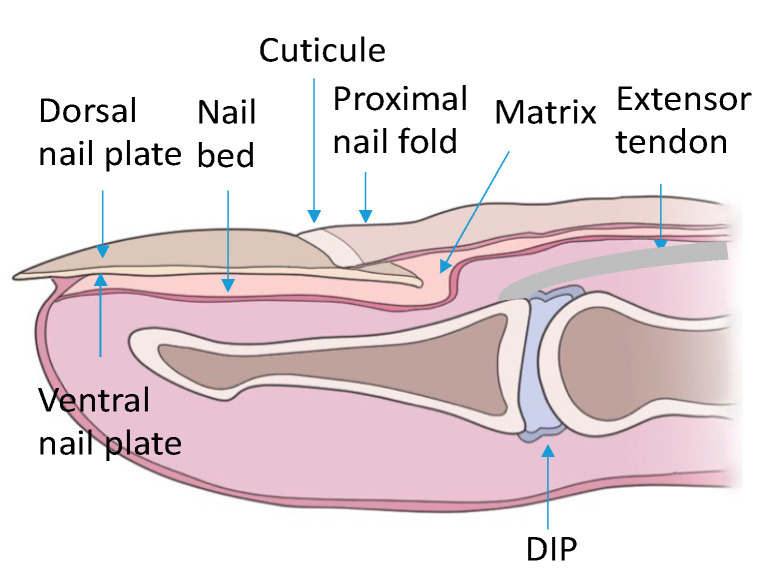
Schematic of the nail apparatus. DIP—distal interphalangeal joint.

The nail apparatus consists of the nail plate, the reproductive part formed by the nail matrix and nail bed, and the soft tissues surrounding the nail plate (Figure 1).

The nail plate is a rather hard yet flexible structure consisting of three layers: two parallel plates (dorsal and ventral), separated by an intermediate space. The nail plate is made up of tile-like adherent keratinocytes. Many factors influence the thickness of the nail plate. The thickness of the nail plate increases with age, it is greater in men than in women. It also depends on the examined finger—the thumb is the thickest and the thickness decreases towards the little finger [14,15].

The nail matrix is located in the proximal part of the nail bed, visible on the nail as a white rim. It is composed of three parts: dorsal, intermediate, and ventral. The main function of the matrix is the growth and formation of the nail plate. The matrix is composed primarily of active keratinocytes. Intensive matrix cell proliferation and keratinization, associated with the removal of parts of the cell nuclei and an increase in cell membrane thickness, are associated with nail plate growth [14,15].

The nail bed is a highly vascularized pink-colored structure, visible under the nail plate. The nail bed is located directly under the nail plate and extends to the periosteum of the distal phalanx. The epidermis of the nail bed is an extension of the ventral part of the matrix. The dermal papillae located under the thin basement membrane of the nail bed epidermis, by forming long appendages, participate in attaching the nail bed to the nail plate, and the numerous collagen fibers present in the nail bed attach it to the periosteum of the distal phalanx [14,15].

## 4. Psoriatic Nail Changes

**Figure 2:**
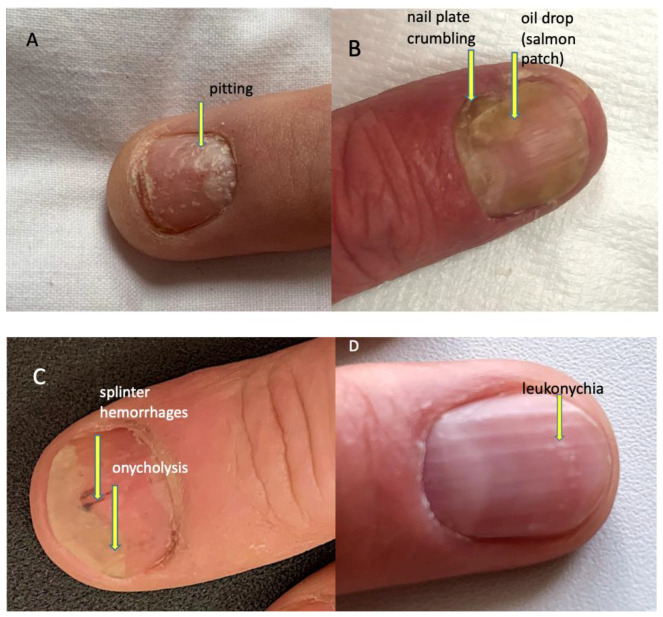
Psoriatic nail changes. (**A**) Pitting (superficial depression within nail plate), (**B**) nail plate crumbling and oil drop/salmon patch (focal parakeratosis leading to focal onycholysis presenting as translucent yellow-red discoloration), (**C**) splinter haemorrhages (longitudinal red-brown splinter shaped haemorrhages under the nail) and onycholysis (detachment of the nail plate from the nail bed presenting as a white-yellow area at the distal part of the nail plate), (**D**) leukonychia (parakeratosis within the nail plate presenting as white spots on the nail).

The evaluated psoriatic nail changes depend on the structure of the nail apparatus which is involved in the disease process. The following are associated with matrix involvement: thinning—small, superficial depressions in the nail plate, leukonychia—small, white spots in the nail plate, red spots in the rim, nail plate dystrophy characterized by increased brittleness and crumbling of the nail and Beau lines—transverse depressions in the plate. In the course of the psoriatic nail bed involvement, they are present: onycholysis—separation of the nail plate from the nail bed, oil spots (salmon spots)—brown-red discoloration under the nail plate, linear subungual hemorrhages, and subungual hyperkeratosis caused by an accumulation of non-exfoliated cells under the nail plate (Figure 2) [16]. The inflammatory process may extend beyond the nail plate to the proximal and lateral nail shafts.

A simple cumulative NAPSI index, based on the assessment of changes from the nail bed and matrix, is often used to clinically assess psoriatic nail changes. The index value is calculated on the basis of its presence in each quadrant of the nail changes which are associated with nail bed and matrix involvement. One point for each nail is awarded for the presence of at least one of the features of matrix involvement: thinning, leukonychia, plate fragility, red spots within the rim, and at least one of the features of nail bed involvement: onycholysis, linear extravasation, hyperkeratosis, oil spot sign. The maximum value of the index for one finger can be 8 points, for all fingers 80 points or 160 if toenails are included. In clinical practice, a modified NAPSI index is also used, which additionally assesses the severity of changes on a scale from 0 to 3 [7,8].

## 5. Ultrasonographic Image of the Nail Apparatus

The nail is a structure consisting of two parallel hyperechoic (white) plates: dorsal and ventral. The plates are separated from each other by a hypoechoic interlaminar space. The thickness of a normal plate varies between 0.3 and 0.65 mm [17].

The nail matrix is an isoechogenic (light grey) structure in the proximal part of the nail, 1–5.3 mm long [12].

The thickness of the hypoechoic (dark gray) nail bed of a healthy nail, measured between the ventral nail plate and the periosteum of the distal phalanx, is 0.7–6.5 mm [17].

**Figure 3:**
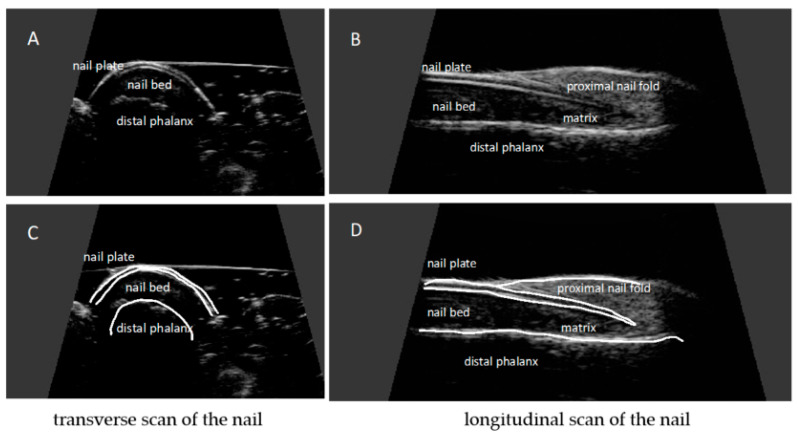
Ultrasonographic image of a healthy nail: transverse (**A**,**C**) and longitudinal view (**B**,**D**). Nail plates presented as a structure consisting of two parallel hyperechoic plates separated by a hypoechoic interlaminar space. Nail matrix presented as an isoechogenic structure in the proximal part of the nail. Nail bed presented as a hypoechoic structure between the ventral nail plate and the periosteum of the distal phalanx.

Under the nail bed, a hyperechoic band is clearly visible on ultrasound, which is the dorsal surface of the distal phalanx. In the proximal part of the dorsal surface of the distal phalanx, a site of attachment of the finger extensor tendon is located (Figure 3).

The ultrasound images included in this article were acquired by using a US system DermaMed (DRAMIŃSKI, Olsztyn, Poland) with a linear head with a frequency of 24–48 MHz.

## 6. Examination Technique

For ultrasonographic examination of the nail apparatus, a high-frequency linear head, above 15 MHz, is used [11,12,13]. Fingernail examination is performed in a seated position with hands placed on a table. The hand nail apparatus is assessed in the sagittal plane, from the dorsal side of the nails, usually in the longitudinal section. It is not recommended to use gel pads to avoid pressure on superficial tissues—the proper distance of the head from the skin, allowing imaging of superficial structures, is maintained only with an appropriate amount of gel. The following parameters are measured: nail plate thickness, nail bed thickness, nail matrix thickness, and finger extensor tendon thickness. Nail thickness is measured as the maximum distance between the dorsal and ventral hyperechoic plates of the nail. The thickness of the hypoechogenic nail bed is measured as the maximum distance between the ventral plate of the nail and the edge of the phalangeal bone. The thickness of the isoechogenic area of the nail matrix is measured at the proximal end of the nail bed. Increased vascularization, a sign of inflammation, is assessed with the power Doppler technique. Tendon attachments are assessed in accordance with OMERACT (outcome measures in rheumatology) recommendations on the scale of grayness. The thickness of the extensor tendon is measured at the place where an extensor tendon is attached to the distal phalanx [13,18,19].

## 7. Ultrasonographic Changes of the Nail Apparatus in Psoriasis and Psoriatic Arthritis

High-resolution ultrasonography can accurately image morphological psoriatic changes in the nail apparatus and objectively assess clinical progression, the severity of active inflammation, and response to treatment.

**Figure 4:**
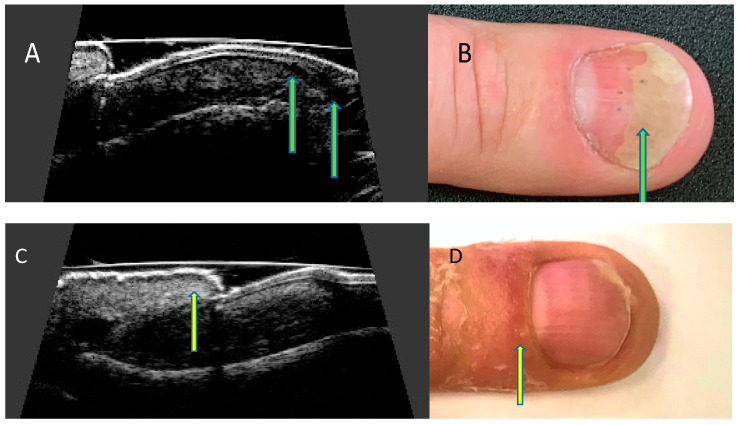
Ultrasonographic changes of the nail apparatus in psoriasis. Onycholysis. (**A**) Separation of the nail plate from the nail bed—ultrasound longitudinal view (arrows), (**B**) separation of the distal edge of the nail bed—nail (arrow). Inflammation of the posterior nail shaft. (**C**) Thickening of the nail shaft—ultrasound longitudinal view (arrow), (**D**) swelling of the nail shaft (arrow).

Ultrasonographic images of nails with psoriatic changes depend on the nature of clinical changes (Figure 4).

Most importantly, it is possible to visualize early psoriatic nail changes on ultrasound images: subclinical (thickening of the nail bed) or with little clinical advancement (loosening of the borders of the ventral plate). Late changes include thickening of the ventral and dorsal nail plate [20]. The activity of inflammation within the matrix and nail bed, associated with increased vascularization of the involved structures, is assessed with the PD technique, on a scale from 0 to 3, where 0 means no PD signal, 1—signal present on less than 25% of the examined area, 2—signal present on more than 25% but less than 50% of the examined area, and 3—signal present on more than 50% of the examined area [21].

The Brown University Nail Enthesis Scale (BUNES) has been suggested for quantitative assessment of ultrasonographic changes of the nail apparatus and inflammatory activity [20]. In the BUNES scale, the presence of psoriatic changes within the nail plate, thickening of the nail bed and thickening of the matrix are scored: 0 for absent ultrasound changes or 1 for the presence of changes (total maximum 3 points), additionally, PD signal in the bed and matrix areas is scored according to the previously described classification (maximum 6 points) [21]. However, the usefulness of the BUNES score, which may prove to be a promising assessment tool in everyday practice, requires further study.

**Figure 5:**
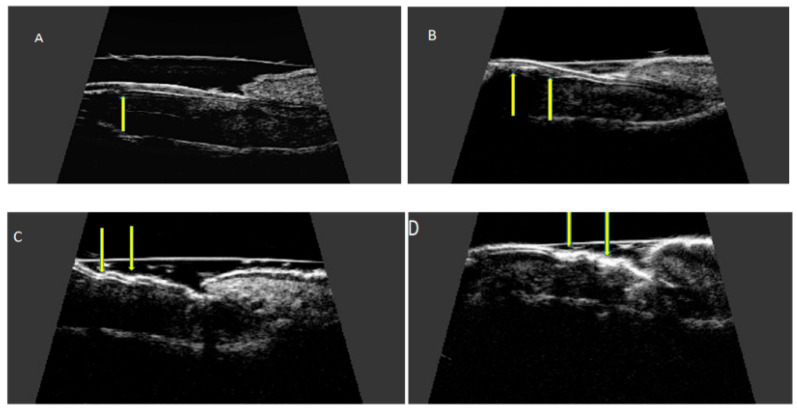
Ultrasonographic changes of the nail plate in nail psoriasis according to Wortsman classification. Longitudinal view. (**A**) focal hyperechoic involvement of the ventral plate—type I, (**B**) loss of the borders of the ventral plate—type II, (**C**) wavy plates—type III, and (**D**) loss of definition of both plates—type IV.

The qualitative severity of psoriatic nail changes is most commonly assessed ultrasonographically according to the classification presented by Wortsman et al. [18] as: focal, point-like hyperechoic involvement of the ventral plate (type I), continuous loss of the borders of the ventral plate (type II), wavy plates—without blurring of both plates (type III) and loss of definition of both plates (type IV) (Figure 5).

**Figure 6:**
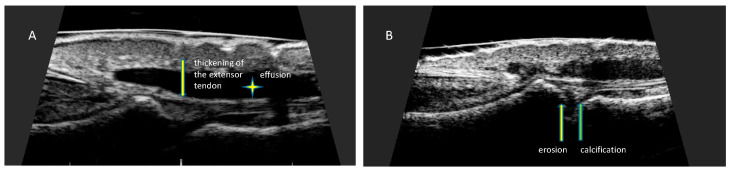
Changes in finger extensor tendon at the place of attachment to the distal phalanx. Longitudinal scan of the finger extensor tendon at the place of attachment to the distal phalanx of the hand. (**A**) Thickening of the tendon at the site of insertion (arrow). Effusion presented as hypoechoic fluid around tendon sheath (asterisk), (**B**) erosion presented as a localized bone loss at the site of tendon insertion (yellow arrow). Calcification presented as hyperechoic structure in the area of the distal attachment of the extensor tendon (green arrow).

Grayscale assessed changes of the finger extensor tendon enthesopathy are consistent with enthesopathy of other sites and include thickening of the tendon, loss of its normal fibrous structure, presence of calcifications, presence of enthesophytes at the attachment site, and osseous changes including erosions (Figure 6).

Ultrasonographic studies in patients with nail psoriasis show significantly increased thickness of nail plates, nail bed, and nail matrix with psoriatic changes present compared to that of the control group [22,23]. Increased nail plate and matrix thickness are observed in psoriatic patients, both with and without psoriatic nail changes present [13,23,24]. In another study, nail plate thickness was significantly greater in patients with Ps/PsA than in those with rheumatoid arthritis (RA) and osteoarthritis (OA), which may be of some value in the differential diagnosis [25]. In a study by Ally Essayed et al., the nail plate thickness of the thumb and index finger above 0.63 mm and 0.61 mm, respectively, were identified as a sign of nail psoriasis (sensitivity 72% and 60% and specificity 70% and 88%, respectively). The thickness of the thumbnail bed above 1.85 mm (sensitivity 64% and specificity 72%) had a similar diagnostic significance in this study. Nail bed thickness of the index fingernail above 1.89 had equally high sensitivity (64%) but much lower specificity (34%) [26]. Another study identified nail bed thickness above 2.0 mm as a cut-off point for the diagnosis of psoriatic changes [27].

Psoriatic nail changes are a known risk factor for the development of psoriatic arthritis [3,28]. In the few studies conducted, ultrasonographic features of finger extensor tendon enthesopathy at the attachment to the distal phalanx were present in more than 50% (52% to 61%) of psoriatic arthritis patients with psoriatic nail changes [22,29]. In patients without arthritis with nail psoriasis, the prevalence of finger extensor tendon enthesopathy was estimated to be between 38% and 60% [22,29]. Increased extensor tendon thickness has been observed in patients with nail psoriasis both among those with and without arthritis [20], an association of tendon thickness with NAPSI score [30], and with skin thickness assessed over the distal interphalangeal joint [24]. In a study by Idolazzi et al., increased vascularization within the extensor tendon attachment visualized by the PD technique was helpful in differentiating inflammatory changes in PsA from osteoarthritis [25]. Surprisingly, in this study, there were no differences in nail bed vascularization in patients with Ps, PsA, RA, and OA, although PD signal was increased in these groups compared with healthy controls. The authors explained this condition by various possible causes of nail bed hypervascularization, including local effects of pro-inflammatory cytokines and biomechanical changes within the nail apparatus.

The treatment of psoriatic nail changes is extremely challenging for clinicians. The therapy of nail psoriasis, in addition to topical treatment, includes systemic retinoids, methotrexate, and cyclosporine [31], as well as biologics and synthetic targeted disease-modifying drugs [32]. In daily practice, the improvement achieved during treatment is assessed entirely on the basis of the clinical manifestation. Ultrasonographic examination of the nails, as an imaging method, can become a tool for objective assessment of the effectiveness of the applied treatment. Few published studies have used the US to monitor treatment efficacy. In one study, after six months of treatment with methotrexate in patients with nail psoriasis with features of enthesopathy present (with and without arthritis), ultrasonography showed a reduction in nail plate, bed and matrix thickness. In both groups (with and without arthritis), methotrexate treatment was associated with a reduction in increased vascularization, assessed with PD at the sites of the tendon attachments examined. In patients without arthritis, methotrexate treatment was associated with a reduction in extensor tendon thickness, whereas no such effect was found in patients with psoriatic arthritis [33]. This may be related to the more frequent occurrence of chronic changes in these patients, which include, in addition to thickening of the tendon resulting from an active inflammatory process, also tendon remodeling with loss of fibrous structure, and the presence of osteophytes and other bony changes in the attachment sites. Such solidified changes may not be sensitive to methotrexate, in contrast to active inflammatory changes in psoriasis. In another study, after six months of acitretin treatment, a reduction in matrix and bed thickness was observed in patients with nail psoriasis, whereas treatment had no effect on extensor tendon thickness or a reduction in increased vascularization in the area of the extensor tendon attachment. This may indicate, on the one hand, the ineffectiveness of treatment of subclinical enthesopathy of the nail apparatus with acitretin and, on the other hand, the potential usefulness of US examination of the nail apparatus in selecting therapy in patients with ultrasonographic features of enthesopathy [34]. In the study by Molina-Leyva et al. patients with Ps were assessed at baseline and at 12, 24, and 52 weeks of the treatment with adalimumab. In this study, a progressive and sustained reduction of vessel resistance was observed at week 52 [35].

**Figure 7:**
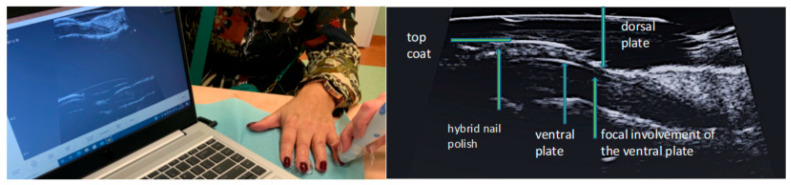
Ultrasound examination of nails with hybrid polish.

In addition to functional disorders, psoriatic nail changes are associated with reduced aesthetics, so many patients try to camouflage the changes with various types of nail polishes, including popular permanent hybrids that cannot be easily removed before examination without destroying the dorsal plate of the nail. In practice, it seriously complicates objective clinical assessment. The dorsal nail plate, which is covered with a layer of polishes, is not visible in the US—the only area in which the dorsal plate of the nail can be visualized is the proximal part of the nail—under the cuticle and deeper under the posterior nail shaft. However, ultrasound imaging allows accurate visualization of the internal structures of the nail apparatus: the ventral plate, the bed, the matrix, the attachment of the finger extensor tendon, also in nails covered with nail polish (Figure 7).

The ultrasound technique is a promising imaging method in nail psoriasis, but it has some significant limitations. Contrary to the principles of joint examination, there is a lack of standardization in the ultrasound technique for the assessment of the nail apparatus. The usefulness of nail ultrasonography as a tool for monitoring disease activity and treatment efficacy in nail psoriasis has been relatively poorly studied. Due to the methodological variability of the studies conducted so far, comparability seems to be significantly difficult (Table 1). Ultrasound examination has not yet been validated in the diagnosis of nail psoriasis, assessment of disease activity, and treatment outcomes. This is a significant obstacle in the use of ultrasound in clinical practice and clinical trials.

## 8. Conclusions

Ultrasonographic examination of the nail apparatus is currently very rarely performed in everyday practice, however, conducted studies indicate the potential usefulness of US in the morphological assessment of the internal structures of the apparatus, including subclinical changes preceding the clinical manifestation of nail psoriatic changes, enthesopathies both subclinical and in the course of psoriatic arthritis and in the assessment of treatment efficacy, as well as in the differentiation of PsA from RA and OA. Undoubtedly, the usefulness of US examination of the nail apparatus in psoriasis requires further study.

## Figures and Tables

**Table 1:** Studies on the evaluation of nails in psoriasis using ultrasound.

Study	Population	Study Design	Imaging Sites	Equipment
De Rossi et al. (2021) [36]	35 PsO, 31 PsA, and 35 controls patients	Cross-sectional	bilateral 2nd and 3rd fingernails	MyLab 50 system (Esaote Biomedica, Genova, Italy), with a linear 10 to 18 MHz transducer and a 6.6 to 8.0 MHz transducer for power Doppler.
Krajewska-Włodarczyk et al. (2021) [34]	41 patients with nail Ps (without PsA), 28 HCs	Prospective	All fingernails	DermaMed (DRAMIŃSKI, Olsztyn, Poland), with linear probe with a frequency of 24 MHz. MyLab Omega (Esaote, Genova, Italy) and with the power Doppler (PD) technique
Mendonça et al. (2020) [37]	30 patients with PsO and PsA (15 for each disease).	Cross-sectional	All fingernails	Esaote MyLab 50, 18 MHz linear probe, power Doppler frequency of 6.6–8 MHz, pulse repetition frequency that varied from 0.5 Hz to 1.0 MHz, and low filter
Idolazzi et al. (2019) [25]	51 patients with PsA, 31 with Ps, 37 with RA, 34 with OA, 50 HCs	Cross-sectional	the middle third of the second fingernail, dominant side hand.	General Electric Logiq S8 machine or Esaote MyLabClassC with a multifrequency linear probe with a frequency of 18 MHz. The power Doppler parameters: a pulse repetition frequency (PRF) of 600 KHz and frequency of 10 MHz
Krajewska-Włodarczyk et al. (2019) [22]	41 patients with Ps and 31 with PsA, 30 HCs	Cross-sectional	All fingernails	DermaMed (DRAMIŃSKI, Olsztyn, Poland). The US nail examinations were conducted with a linear head with a frequency of 24 MHz.
Moreno et al. (2019) [38]	35 patients with Ps onychopathy and 25 with nail dystrophy secondary to onychomycosis	Cross-sectional	One (most affected) nail	Esaote My Lab 60© Ultrasound System (Esaote, Genova, Italy), transducer frequency range of 7–13 MHz, equipped with Doppler
Naredo et al. (2019) [39]	60 patients with PsA, 21 with PsO, and 20 HCs	Cross-sectional	All fingers of both hands	Esaote Mylab Twice, (Genoa, Italy) equipped with a multifrequency (10–22 MHz) linear transducer
Bakirci et al. (2018) [40]	34 patients with nail Ps and 15 HCs	Cross-sectional	right second finger.	LOGIQ P9 (General Electric Company, United Kingdom), with a 7–13 MHz linear transducer
Moya Alvarado et al. (2018) [30]	48 patients with Ps and asymptomatic PsA (25 Ps, 23 PsA)	Prospective	Five nails of the dominant hand	MyLab Touch, Esaote Biomedica, Italy) with a variable frequency transducer with a linear array of 18 to 22 MHz in mode B
Mondal et al. (2018) [41]	45 patients with PsA and 45 HCs	Case-control	All fingernails	My Lab 25 gold, Esaote, with 18 MHz linear array transducer
Krajewska-Włodarczyk et al. (2018) [22]	69 patients with psoriatic changes in nails (38 with Ps and 31 with PsA) and 30 HCs	Cross-sectional	All fingernails	DermaMed (DRAMIŃSKI, Olsztyn, Poland) with a linear head with a frequency of 24 MHz.
Krajewska-Włodarczyk et al. (2018) [33]	32 patients with nail Ps and with DIP joint extensor tendon enthesopathy in at least one finger revealed in a US examination (19 with Ps and 13 with PsA)	Prospective	All fingernails	DermaMed (DRAMIŃSKI, Olsztyn, Poland) with a linear probe with a frequency of 24 MHz.
Idolazzi et al. (2018) [42]	82 patients with Ps and/or PsA, and 50 HCs	Cross-sectional	the middle third of the second fingernail, dominant side hand.	General Electric Logiq S8 with a multifrequency linear probe (Li8-18) with setting at 18 MHz. Power Doppler parameters were set selecting a PRF of 600 KHz and frequency of 10 MHz.
Molina-Leyva et al. (2018) [35]	15 patients with moderate-severe Ps	Prospective	proximal nailfold of the fourth finger of the nondominant hand	n/a
Acosta-Felquer et al. (2017) [29]	54 patients with PsO and 56 with PsA	Cross-sectional	All fingernails	MyLab 70 (Esaote Biomedica, Genoa, Italy) linear head (6–18 MHz)
Acquitter et al. (2017) [43]	18 nail PsO, 19 scalp or inverse PsO	Prospective	Every patient was scanned for 14 entheses and 12 nails (10 fingernails and 2 toenails)	IU 22 machine (Philips) linear probe at 12.5 MHz.
Arbault et al. (2016) [44]	27 patients with PsA	Pilot prospective study	All fingernails	ESAOTE MyLab 70 XVG fitted with a high frequency transducer of 22 mHz
Marina et al. (2016) [45]	23 patients with moderate-to-severe psoriasis, (14 with nail psoriasis and 9 without nail involvement), and 11 HCs	Cross-sectional	79 fingernails with Ps changes, 43 fingernails without Ps changes 82 fingernails in HCs	Ultrasonix Medical Corporation, (Richmond, Canada) with a variable-frequency (from 8 to 40 MHz) transducer (focal range 0.2–3 cm, image field 16 mm) and Hitachi EUB 8500 System with a variable-frequency transducer (6.5–13 MHz)
Sandobal et al. (2014) [27]	35 patients with PsA, 20 with Ps, and 2 control groups (28 control subjects and 27 patients with RA)	Cross-sectional	All nails of both hands	Esaote Biomedica, Genoa, Italy) with a variable-frequency transducer ranging from 10 to 18 MHz and a Doppler frequency ranging from 6 to 8 MHz
Aydin et al. (2013) [5]	5 patients with PsO, 13 with PsA with at least one involved nail, 12 healthy controls	Cross-sectional	All fingernails	Logiq E9 machine (General Electric, Wauwatosa, Wisc., USA) with a linear probe at 9–14 MHz
Aydin et al. (2012) [24]	86 Ps patients and 20 healthy controls	Cross-sectional	2 fingernails (one on each hand)	Logiq E9 machine (General Electric, Wauwatosa, Wisc., USA) with a linear probe at 10–18 MHz
Gisondi et al. (2012) [23]	138 patients with Ps, 46 healthy controls, 37 with chronic eczema	Cross-sectional	Right hand fingernails	Voluson I portable ultra- sound machine (General Electrics, United States) with linear 10–18 MHz probe equipped with a variable-fre- quency transducer of 18 MHz
Gutierrez et al. (2012) [46]	21 patients with PsA	Prospective	16 joints, 9 tendons, 11 enthesis, 16 psoriatic plaques and 8 psoriatic onychopathies	MyLab 70 XVG (Esaote SpA, Genoa, Italy) with a broadband frequency transducer ranging from 6 to 18 MHz and Doppler frequency ranging from 5.9 to 14.3 MHz
Husein El-Ahmed et al. (2012) [47]	23 patients with moderate-to-severe Ps and 23 controls without Ps	Cross-sectional	Echo Doppler examination on the proximal third of the nail plate of the fourth finger of the nondominant hand.	n/a
Gutierrez et al. (2010) [21]	30 patients with PsA	Cross-sectional	n/a	MyLab 70 XVG US system (Esaote Biomedica Genoa, Italy) with a 6–18 MHz linear transducer (B-mode frequency of 18 MHz and Doppler frequency of 9.1 MHz)
Gutierrez et al. (2009) [48]	30 patients with Ps, 15 HCs	Cross-sectional	Nails with psoriatic changes	MyLab 70 XVG system (Esaote Biomedica, Genoa, Italy) equipped with a variable-frequency transducer ranging from 6 to 18 MHz
Fournié et al. (2006) [O] [49]	21 patients with RA, 20 with PsA	Cross-sectional	25 fingers in RA (1 finger in 18 patients, 2 in 2 patients, and 3 in 1 patient) and 25 fingers in PsA (1 finger in 15 patients and 2 in 5 patients)	Siemens Sonoline Elegra (Cheshire, CT, USA) with 13.5-MHz linear transducer
Wollina et al. (2001) [P] [50]	37 patients with nail diseases (11 with SLE, 8 with systemic sclerosis, 9 with Ps, 5 with chronic hand eczema and others, and 34 healthy controls	Cross-sectional	All fingernails	Derma-scan C, Cortex Technology (Hadsund, Denmark) with a 20 MHz probe in B-scan mode.

Ps: psoriasis, PsA: psoriatic arthritis, HCs: healthy controls, RA: rheumatoid arthritis, OA: osteoarthritis, DIP: distal interphalangeal joint, US: ultrasound, SLE: systemic lupus erythematosus.
